# Technologist primer for MRI right heart catheterization: the NIH and CNMC experience

**DOI:** 10.1186/1532-429X-16-S1-T1

**Published:** 2014-01-16

**Authors:** Jonathan R Mazal, Kendall O'Brien, William Schenke, Annette Stine, Laurie Grant, Anthony Z Faranesh, Kanishka Ratnayaka, Robert J Lederman

**Affiliations:** 1 National Heart Lung and Blood Institute, National Institutes of Health, Bethesda, Maryland, USA; 2 Children's National Medical Center, Washington DC, District of Columbia, USA

## Background

We have begun to perform routine and research MRI right heart catheterization in adults and research MRI catheterization in children. We report our technologist workflow.

## Methods

Scanner preparation: The scanner façade and bore are covered with adhesive clear sterile drapes and surface coils enveloped in sterile plastic bags. A MR catheterization pack contains only non-ferromagnetic supplies. Sterile field and transfer: After vascular access in the adjoining X-ray lab, arterial sheaths are secured, and the catheterization field is covered with a pair of additional sterile drapes that can be folded to allow the surface coils to be repositioned in sterile fashion. Patient connections-- noninvasive BP cuff, ECG electrodes, pulse oximeter, pressure transducers, intravenous lines, oxygen --are connected to the MR system before transfer on a multimodality table. All tubes enter the bore from the same end as the patient to allow rapid evacuation. Imaging: We systematize image acquisition and display order for efficiency and familiarity. Reference images guide real-time acquisition during catheter navigation at specific steps, and include orthogonal views for each of: inferior and superior vena cavae, right ventricular inflow and outflow tracts, main pulmonary artery bifurcation, and both pulmonary artery branches. A standardized imaging protocol also is applied for cardiac function during multiple provocations, triggered by catheterization findings. MR flow is analyzed interactively. Procedural communication: In our experience, open-microphone multi-channel sound-suppression headsets require "audio discipline" to minimize extraneous conversation and miscommunication. Patient evacuation procedure: Emergency evacuation is drilled quarterly. Drills focus on team member roles and on routing devices (coils and tubes) that may tether the patient to the MRI and hinder evacuation. This assures that time to simulated defibrillation on the neighboring X-ray table is less than 60 seconds.

## Results

To date we have peformed over 50 MR right heart catheterization procedures.

## Conclusions

With practice, we have developed efficient and safe workflows for diagnostic MRI catheterization.

## Funding

This work is supported by the Division of Intramural Research (Z01-HL005062-08, Z01-HL006039-01), National Heart Lung and Blood Institute, National Institutes of Health, USA.

**Figure 1:**
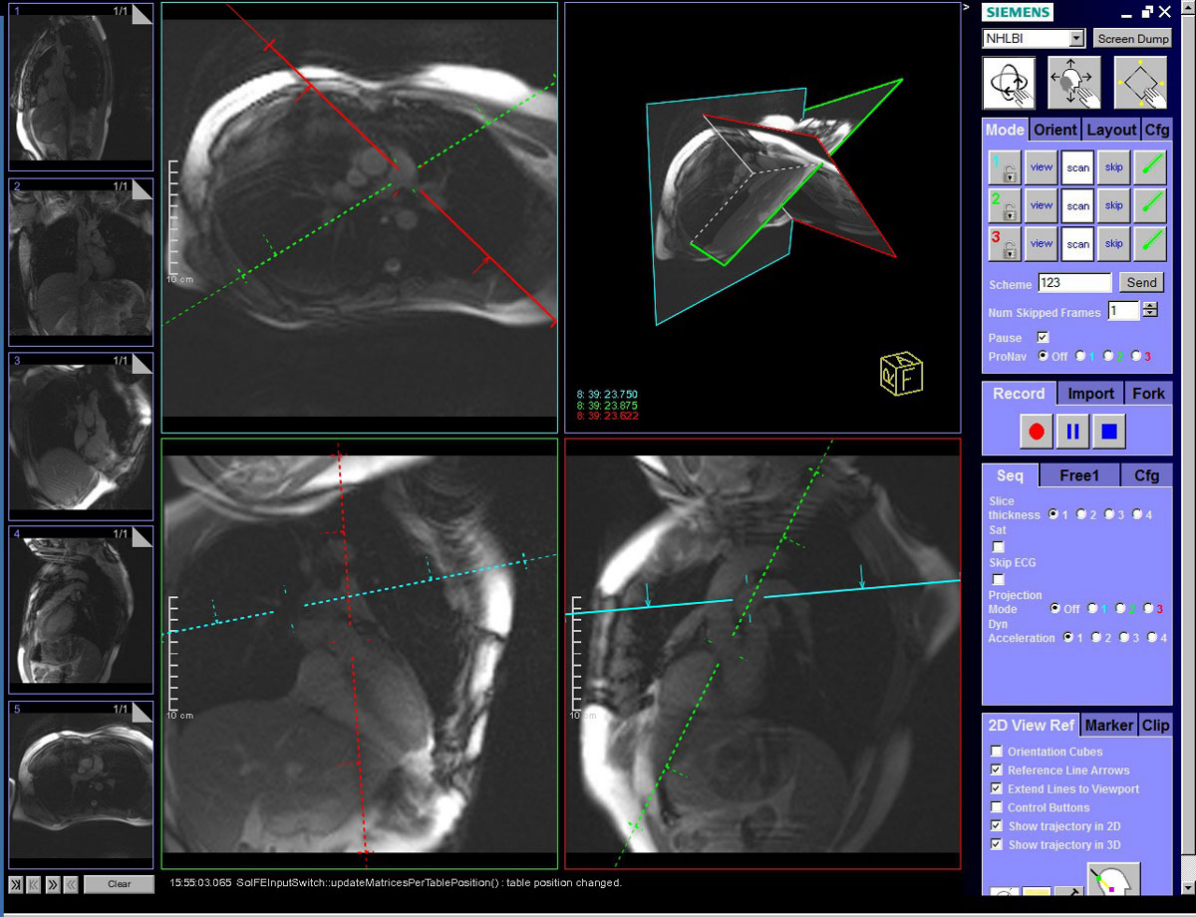
**Planning of reference images for guiding real-time acquisition during catheter navigation via a dedicated user interface (Interactive Front End, Siemens; Erlangen, Germany)**.

**Figure 2:**
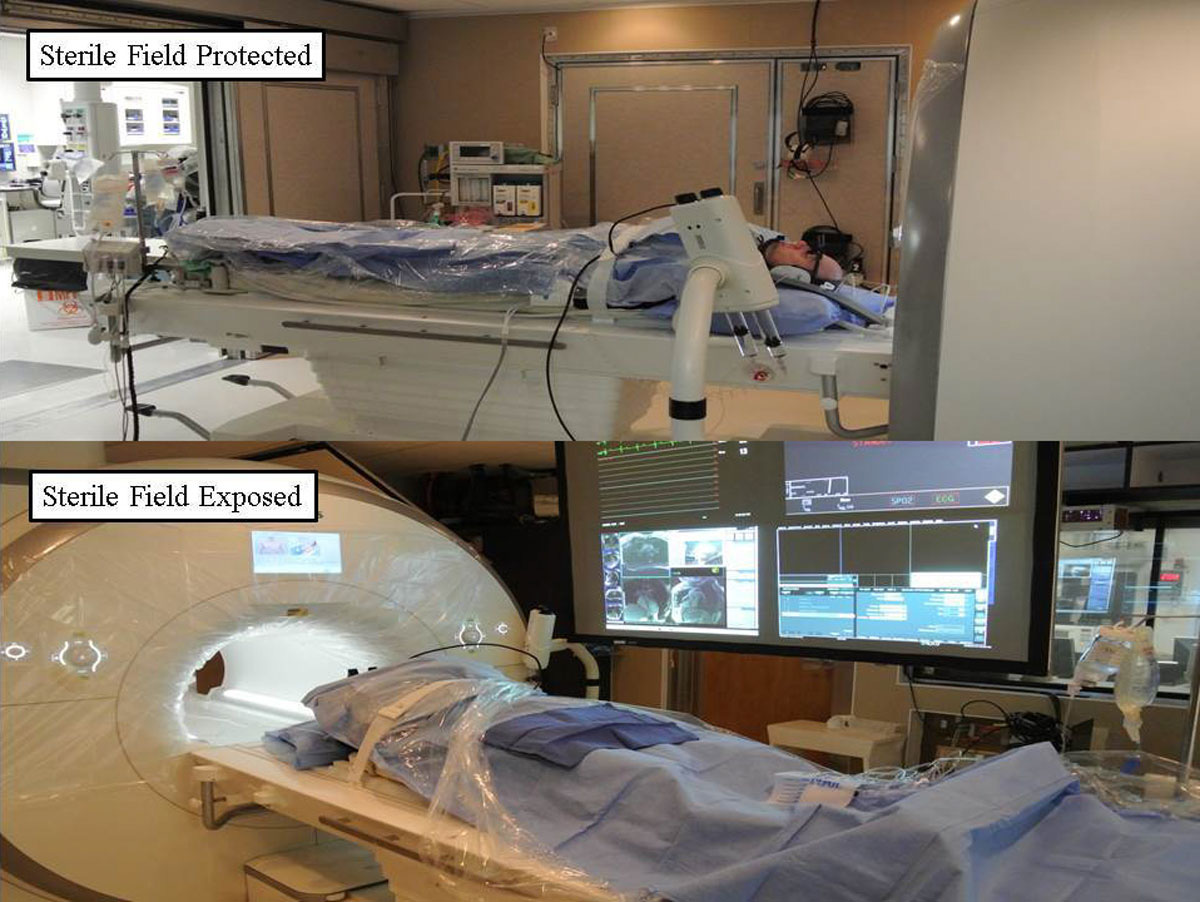
**Demonstration of preparation of the MR scanner room and patient, protecting the sterile field while maintening access to the vascular access site**.

